# The Selenoprotein MsrB1 Instructs Dendritic Cells to Induce T-Helper 1 Immune Responses

**DOI:** 10.3390/antiox9101021

**Published:** 2020-10-20

**Authors:** Ho-Jae Lee, Joon Seok Park, Hyun Jung Yoo, Hae Min Lee, Byung Cheon Lee, Ji Hyung Kim

**Affiliations:** 1 Department of Biosystems and Biotechnology, College of Life Sciences and Biotechnology, Korea University, Seoul 02841, Korea; lhj2894@korea.ac.kr; 2 Department of Immunology, Blavatnik Institute, Harvard Medical School, Boston, MA 02115, USA; joonseok_park@hms.harvard.edu; 3 Department of Biotechnology, College of Life Sciences and Biotechnology, Korea University, Seoul 02841, Korea; 920618mn@korea.ac.kr (H.J.Y.); molhm99@korea.ac.kr (H.M.L.); cheonii@korea.ac.kr (B.C.L.)

**Keywords:** methionine sulfoxide reductase B1, methionine oxidation, dendritic cells, T-cell activation, T-helper 1, signal transducer and activator of transcription-6 (STAT6)

## Abstract

Immune activation associates with the intracellular generation of reactive oxygen species (ROS). To elicit effective immune responses, ROS levels must be balanced. Emerging evidence shows that ROS-mediated signal transduction can be regulated by selenoproteins such as methionine sulfoxide reductase B1 (MsrB1). However, how the selenoprotein shapes immunity remains poorly understood. Here, we demonstrated that MsrB1 plays a crucial role in the ability of dendritic cells (DCs) to provide the antigen presentation and costimulation that are needed for cluster of differentiation antigen four (CD4) T-cell priming in mice. We found that MsrB1 regulated signal transducer and activator of transcription-6 (STAT6) phosphorylation in DCs. Moreover, both in vitro and in vivo, MsrB1 potentiated the lipopolysaccharide (LPS)-induced Interleukin-12 (IL-12) production by DCs and drove T-helper 1 (Th1) differentiation after immunization. We propose that MsrB1 activates the STAT6 pathway in DCs, thereby inducing the DC maturation and IL-12 production that promotes Th1 differentiation. Additionally, we showed that MsrB1 promoted follicular helper T-cell (Tfh) differentiation when mice were immunized with sheep red blood cells. This study unveils as yet unappreciated roles of the MsrB1 selenoprotein in the innate control of adaptive immunity. Targeting MsrB1 may have therapeutic potential in terms of controlling immune reactions.

## 1. Introduction

Reduction–oxidation (Redox) reactions participate in diverse physiological processes. One of these Redox reactions is protein oxidation, which is achieved by reactive oxygen species (ROS), and plays an important role in regulating cellular signal transduction, metabolism, and protein turnover and activity [1]. This post-translational oxidative modification occurs readily and reversibly on two sulfur-containing amino acids, namely, methionine (Met) and cysteine (Cys), and the Cys analog amino acid called selenocysteine (Sec), in which the sulfur of Cys is substituted by selenium. With regard to Met, the oxidation of its sulfur group generates *S*- and *R*- stereoisomers of methionine sulfoxide. This oxidative action of ROS on Met can be countered by methionine sulfoxide reductase A (MsrA) and B (MsrB) which, respectively, reduce methionine-*S*-sulfoxide and methionine-*R*-sulfoxide (Met-*R*-SO) back to Met [2,3].

There are three mammalian MsrB proteins: MsrB1, MsrB2, and MsrB3. All are able to reduce Met-*R*-SO. Unlike MsrB2 and MsrB3, MsrB1 is a selenoprotein, namely, a protein that contains Sec residues that catalyze Redox reactions [4,5]. Thus, Sec residues play important roles in protein oxidation as both Redox targets and Redox catalyzers. It should be noted that MsrB1 is mainly expressed in the cytosol and nucleus (by contrast, MsrB2 and MsrB3 are only found in the mitochondria and/or endoplasmic reticulum) [6]. Selenium and selenoproteins like MsrB1 have been found to be important for human health [7] but their physiological and cellular roles remain to be established.

ROS also participate in a variety of adaptive and innate immunity processes, sometimes by inducing protein oxidation. An example of the roles of ROS in adaptive immunity relates to T-cells: on antigenic stimulation, T-cells undergo a dramatic metabolic rewiring that induces oxidative phosphorylation; this produces ROS [8], which in turn promote T-cell proliferation and activation [9] and play critical roles in shaping T-cell function and survival [10,11]. An example of the roles of ROS in the innate immune system, which have been particularly well-studied, relate to macrophages and dendritic cells (DCs): when these cells receive signals through their pattern recognition receptors, they upregulate their mitochondrial respiration to generate mitochondrial ROS, which kill the microbes along with cellular ROS generated by membrane-bound nicotinamide adenine dinucleotide phosphate (NADPH) oxidase complexes [8,12]. Notably, there is also evidence that ROS participate in the ability of innate immune cells to regulate adaptive immune cell functions: the cellular ROS produced by antigen-stimulated DCs regulates the pH in their phagosomes, which in turn shapes their antigen presentation and their ability to stimulate T-cell proliferation and cytokine production [13,14,15]. The ability of ROS to shape the antigen processing and presentation of antigen-presenting cells has been actively investigated. However, the mechanism(s) by which ROS mediate innate-to-adaptive immune cell signaling and how these mechanisms are regulated remain less well-explored.

It is possible that one of these ROS-mediated cellular cross-talk mechanisms involves the methionine reductase MsrB1: this is supported by two studies that show that MsrB1 in bone marrow-derived macrophages counters the oxidizing activity of a molecule interacting with CasL protein (Mical) monooxygenase on two Met residues in actin [16]. Thus, while Mical converts these Met residues to Met-*R*-SO, thereby inhibiting macrophage actin filament assembly, MsrB1 reduces Met-*R*-SO back to Met, thereby promoting actin polymerization. In addition, MsrB1 is needed for their lipopolysaccharide (LPS)-induced production of anti-inflammatory cytokines [17]. Potentially, these mechanisms have broader consequences for other immune cells.

The roles of MsrB1 in DCs, which play a central role in antigen presentation and T-cell priming, have not yet been investigated. Here, we showed with MsrB1-deficient mice and bone marrow-derived DCs (BMDCs) that MsrB1 in DCs controls adaptive immune responses both in vitro and in vivo. Specifically, we showed that MsrB1 in DCs promotes (i) DC maturation and antigen presentation to and costimulation of naïve cluster of differentiation antigen four (CD4) T-cells, (ii) DC production of Interleukin-12 (IL-12), (iii) signal transducer and activator of transcription-6 (STAT6) activation in DCs, (iv) DC-induced antigen-specific T-helper 1 (Th1) cell differentiation, and (v) follicular helper T (Tfh) cell differentiation in vivo. We propose that Met reduction by MsrB1 may control STAT6 phosphorylation, which in turn activates the maturation and T-cell-stimulatory functions of DCs.

## 2. Materials and Methods

### 2.1. Animal Experiments

MsrB1 deficient (MsrB1^−/−^) mice on C57BL/6J background were generated as previously reported [18]. Wild-type C57BL/6J mice were purchased from OrientBio (Sungnam, Korea). OT-I and -II mice, which, respectively, produce major histocompatibility complex (MHC) class I- and II-restricted transgenic chicken ovalbumin (OVA)-specific CD8 and CD4 T-cells [19,20], were kindly provided by Dr. Se Ho Park and Dr. Tae Sung Kim in the Life Science Division of Korea University, Seoul, Korea. Sex- and age-matched animals between 8 and 12 weeks of age were used for all experiments. Mice were maintained in a specific pathogen-free animal facility at Korea University. All murine experiments were performed according to the guidelines of the Korea University Institutional Animal Care and Use Committee (KUIACUC-2019-0006, KUIACUC-2019-0107).

### 2.2. Generating Splenocyte Preparations

To obtain splenic cells in suspension, spleens from mice were cut into small pieces and incubated for 30 min (min) at 37 °C in a digestive solution consisting of Roswell Park Memorial Institute (RPMI) 1640 medium (HyClone, Logan, UT, USA), 5 mg/mL Collagenase IV (Worthington, Columbus, OH, USA), and 10 μg/mL DNase I (Sigma, St. Louis, MO, USA). The reaction was stopped by adding cell culture medium consisting of RPMI 1640 medium supplemented with 100 U/mL penicillin–streptomycin (Gibco, Carlsbad, CA, USA), 2 mM sodium pyruvate (Gibco, Carlsbad, CA, USA), 10 mM hydroxyethyl piperazine ethane sulfonicacid (HEPES) (Gibco, Carlsbad, CA, USA), and 10% heat-inactivated fetal bovine serum (FBS) (HyClone, Logan, UT, USA). The spleens were then passed through a 70 μm cell strainer (SPL Life Science, Pocheon-si, Korea) and centrifuged at 350× *g* for 5 min. The red blood cells were lysed with red blood cell (RBC) lysis buffer for 5 min and the splenic cell preparation was washed twice by fresh cell culture medium, and 100 ng/mL LPS isolated from *Escherichia coli* 0111:B4 (Millipore Sigma, Burlington, MA, USA) was added for the indicated durations.

### 2.3. Generating Bone Marrow-Derived Dendritic Cell Cultures

To obtain BMDCs, the bone marrow was flushed from the femur and tibia, and clusters within the bone marrow suspension were dispersed by vigorous pipetting. After red blood cell (RBC) lysis using RBC lysis buffer, the cells were washed twice with fresh cell culture medium. The cells were seeded with 20 ng/mL granulocyte-macrophage colony-stimulating factor (GM-CSF) (BioLegend, San Diego, CA, USA) into 100 mm Petri dishes at a concentration of 1 × 10^6^ cells/mL. On days 3 and 6, half of the culture medium was replaced with fresh cell culture medium containing 20 ng/mL GM-CSF. In some experiments, the BMDCs were generated with 10 ng/mL of IL-4 (BioLegend, San Diego, CA, USA) and 20 ng/mL GM-CSF rather than GM-CSF alone. For all experiments, the BMDCs were harvested on day 8. To induce BMDC maturation, the BMDCs were replated in 6- or 24-well plates at 1 × 10^6^ cells/mL in fresh cell culture medium, and 100 ng/mL LPS (Sigma, Burlington, MA, USA) was added for the indicated durations.

### 2.4. In Vitro Stimulation of OT-II Cells with OVA-Pulsed BMDCs

BMDCs from wild-type (WT) or MsrB1-deficient mice were harvested 8 days after their isolation and culture, and pulsed with 0, 10, 25, or 50 μg/mL of peptide-free OVA grade VII (Sigma, Burlington, MA, USA) for 18 h. To obtain single cell suspensions from OT-II mouse, spleens were passed through a 70 μm cell strainer, and red blood cells were lysed with RBC lysis buffer. Lymph nodes were incubated in the digestive solution for 30 min at 37 °C and excised using needles, followed by being passed through a 70 μm cell strainer. The OT-II CD4^+^ T-cells were enriched by using a mouse CD4 T-cell isolation kit (Miltenyi Biotec, Bergisch Gladbach, Germany) and separation on magnetic-activated cell sorting (MACS) LS columns (Miltenyi Biotec, Bergisch Gladbach, Germany). The OT-II CD4^+^ T-cells were then stained with 5 μM carboxyfluorescein succinimidyl ester (CFSE) (BioLegend, San Diego, CA, USA) in phosphate buffered saline (PBS) at 37 °C for 5 min and cocultured in round-bottom 96-well plates with the OVA-pulsed DCs at a DC/T-cell ratio of 2 × 10^4^:2 × 10^5^ (1:10). T-cell proliferation (CFSE dilution) and activation (CD25 and CD44 expression) were analyzed by flow cytometry.

### 2.5. Western Blot

BMDCs (or bone marrow-derived macrophages, which were generated as described above for BMDCs except macrophage colony-stimulating factor (M-CSF) was used) were lysed in protease inhibitor- and phosphatase inhibitor-containing CelLytic M buffer (Sigma, Burlington, MA, USA) and 20 μg of the cell lysates were separated via 15% or 7.5% SDS-PAGE, transferred to a polyvinylidene fluoride (PVDF) membrane, stained with primary antibodies, incubated with horseradish peroxidase (HRP)-labeled Immunoglobulin G (IgG) (Bio-Rad, Hercules, CA, USA), and visualized with enhanced chemiluminescence (ECL) clarity substrate (Bio-Rad, Hercules, CA, USA). The primary antibodies were anti-MsrB1 (LF-PA0088, 1:1000 dilution, Invitrogen, Carlsbad, CA, USA), anti-STAT6 (ab32520, 1:2000 dilution, Abcam, Cambridge, UK), antiphospho-STAT6 (D8S9Y, 1:1000 dilution, Cell Signaling Technology, Danvers, MA, USA), anti-β-actin (sc-47778, 1:5000 dilution, Santa Cruz, CA, USA), and anti-α-tubulin (T5168, 1:5000 dilution, Sigma, Burlington, MA, USA).

### 2.6. RNA Extraction and Quantitative Real-Time PCR (qPCR)

Total RNA was extracted by using TRIzol (Invitrogen, Carlsbad, CA, USA). The RNA was converted to cDNA by using a high capacity RNA-to-cDNA kit (Applied Biosystems, Foster City, CA, USA). The cDNA was mixed with primers and iTaq universal SYBR Green supermix (Invitrogen, Carlsbad, CA, USA) and relative expression was determined by real-time PCR. *Ppia* was used as a housekeeping gene. To calculate the relative fold change, the two-cycle threshold method was used. The primer sequences are listed in Table 1.

### 2.7. Flow Cytometry

Cells were stained with antibodies against surface antigens in fluorescence-activated cell sorting (FACS) buffer (PBS supplemented with 0.5% BSA) at 4 °C for 20 min. Subsequently, the cells were washed and analyzed by using a Cytoflex (Beckman, Guangzhou, China) and FlowJo (Becton, Dickinson and Company (BD), Franklin Lakes, NJ, USA). Propidium iodide (BioLegend, San Diego, CA, USA), or Zombie Aqua dye (BioLegend, San Diego, CA, USA) was used to exclude dead cells in all FACs analyses. For intracellular cytokine staining, cells were stimulated in cell culture medium for the indicated times at 37 °C in a CO_2_ incubator and Brefeldin A (BioLegend, San Diego, CA, USA) was added 4 h before harvest. To detect interferon-γ (IFN-γ), the cells were stimulated with 20 ng/mL of phorbol 12-myristate 13-acetate (PMA) and 1 μg/mL of ionomycin. After surface staining, the cells were fixed and permeabilized with a Cyto-Fast solution (BioLegend, San Diego, CA, USA) followed by staining with cytokine-specific antibodies in wash buffer. The FACs antibodies are listed in Table 2.

To detect pSTAT6, intracellular phosphoprotein flow cytometry was performed [21]. In brief, cells were stained for surface markers, fixed with 4% paraformaldehyde, washed, incubated in ice cold methanol for 10 min, washed with PBS, and incubated first with antiphospho-STAT6 (D8S9Y, 1:100 dilution, Cell Signaling Technology, Danvers, MA, USA) and then with Alexa647-conjugated anti-rabbit IgG (Thermo Fisher, Waltham, MA, USA) in staining buffer.

### 2.8. LPS Challenge, Adoptive Transfer, and Sheep Red Blood Cell Immunization

For the LPS challenge experiments, mice were injected with 50 μg of LPS intraperitoneally. After 4 h, the splenocytes were obtained and analyzed for DC maturation and T-cell activation. For the adoptive transfer experiments, 1 × 10^6^ of CFSE-labeled OT-II CD4^+^ T-cells were injected intravenously into recipient mice. After 1 day, the recipient mice were immunized with 15 μg OVA and 15 μg LPS mixed in PBS by intraperitoneal injection. Splenocytes were isolated from the mice 3 days later and analyzed for OT-II cell activation and proliferation. For sheep RBC (SRBC) immunization, 100% packed SRBCs (Innovative Research, Novi, MI, USA) were washed and resuspended in PBS, and 2 × 10^8^ SRBCs were injected intraperitoneally into mice. A week later, the splenocytes were isolated and analyzed for follicular helper T-cells.

### 2.9. Enzyme-Linked ImmunoSorbent Assay (ELISA)

To detect the levels of IL-12p70 and IL-23p19 in the cultured media, ELISA was performed using the IL-12p70 ELISA kit (BioLegend, 433604, San Diego, CA, USA) and IL-23p19 ELISA kit (Invitrogen, 88-7230-22, Carlsbad, CA, USA), respectively. The supernatants of the BMDC cultures at 0, 12, and 24 h of LPS stimulation were subject to ELISA assay following the manufacturer’s instructions.

### 2.10. Statistical Analysis

All data are presented as mean ± standard error. To compare two groups, two-tailed unpaired *t*-test or Mann-Whitney U test were used for the normally and non-normally distributed data, respectively. The number of asterisks represents the degree of significance with respect to *p*-value; the exact value is shown in each figure legend. All statistical analyses were performed with Prism 5 (GraphPad Software, San Diego, CA, USA).

## 3. Results

### 3.1. MsrB1 Promotes DC Maturation

Although Lee et al. examined the mRNA expression of MsrB1 in various cells, including DCs [17], they did not assess the protein levels of this selenoprotein. Therefore, we first examined whether MsrB1 protein is expressed in DCs. Indeed, in the absence of LPS stimulation, MsrB1 was reliably expressed in cultured BMDCs. Moreover, this protein expression was comparable to that in bone marrow-derived macrophages that had been stimulated with LPS for 24 h (Figure S1A).

We then used MsrB1-deficient mice to assess the role of MsrB1 in DC regulation of T-cell functions. The genetic ablation of MsrB1 did not change the in vivo DC phenotype: the ablated mice exhibited a normal distribution of the classical DC subsets in the spleen (Figure S1B). The absence of *Msrb1* gene expression also had no effect on the GM-CSF-induced differentiation of DCs from their bone marrow progenitors (Figure S2).

**Figure 1:**
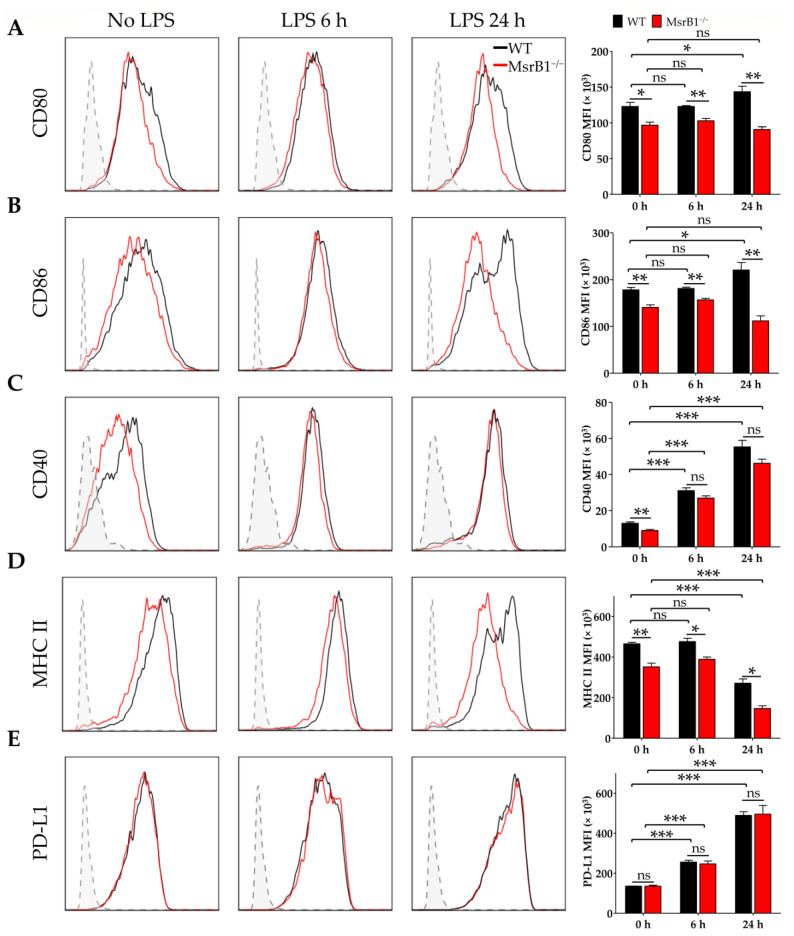
Methionine sulfoxide reductase (Msr)B1 is needed for normal lipopolysaccharide (LPS)-induced maturation of dendritic cells (DCs). Bone marrow-derived DCs (BMDCs) from wild-type (WT) and MsrB1^−/−^ mice were stimulated with LPS for the indicated durations (*n* = 3 per group) and their expression of CD80 (**A**), CD86 (**B**), CD40 (**C**), major histocompatibility complex (MHC) II (**D**), and PD-L1 (**E**) were analyzed by flow cytometry with gating on the CD11c^+^CD11b^+^ BMDCs in the live cells. In all figures, MFI stands for mean fluorescence intensity. Fluorescence minus one (FMO) controls (gray) are shown in each histogram. Mean ± SEM are shown. * *p* < 0.05, ** *p* < 0.01, *** *p* < 0.001; ns, not significant, as determined by unpaired *t-*test. The data shown are from one of four independent experiments, all of which had similar results.

To determine whether MsrB1 can regulate the ability of DCs to present antigen and prime T-cells, we stimulated WT or MsrB1-deficient BMDCs with LPS. LPS normally causes DCs to mature and start expressing T-cell costimulatory molecules such as CD80, CD86, and CD40 [22,23]. Interestingly, the LPS-induced upregulation of CD80 and CD86 in MsrB1-deficient BMDCs was significantly compromised (Figure 1A,B). LPS-induced upregulation of CD40 expression was also slightly (albeit not significantly) reduced by MsrB1 deficiency (Figure 1C). The absence of MsrB1 also significantly reduced the major histocompatibility complex (MHC) class II expression by the BMDCs, particularly 24 h after LPS stimulation (Figure 1D). Notably, unlike the deleterious effect of MsrB1 deficiency on DC costimulatory molecules, MsrB1 knockout had no effect on the BMDC surface expression of programmed death-ligand 1 (PD-L1), which is a well-documented coinhibitory ligand (Figure 1E); this implies that MsrB1 may selectively affect the ability of DCs to stimulate T-cells.

### 3.2. MsrB1 in DCs Controls Antigen-Specific Proliferation of T-Cells In Vitro

**Figure 2:**
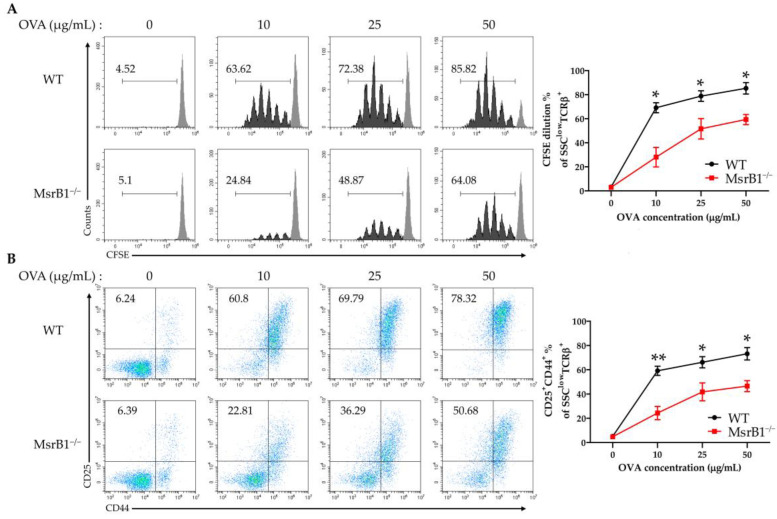
MsrB1 is needed for the ability of DCs to induce CD4 T-cell proliferation and activation. carboxyfluorescein succinimidyl ester (CFSE)-stained OT-II cells were cocultured for 3 days with WT and MsrB1^−/−^ BMDCs that had been loaded with 0, 10, 25, or 50 μg/mL ovalbumin (OVA) (*n* = 3 per group). The CFSE dilution (**A**) and CD25 and CD44 expression (**B**) of the OT-II cells were measured by flow cytometry with gating on the side scatter (SSC)^low^ T cell-receptor (TCR)-β^+^ cells in the live cells. Mean ± SEM are shown. * *p* < 0.05, ** *p* < 0.01; ns, not significant, as determined by unpaired *t-*test. The data are representative of two independent experiments, both of which had similar results.

The fact that the LPS-treated MsrB1-deficient DCs expressed lower levels of costimulatory molecules and MHC class II suggests that MsrB1 may participate in DC priming of CD4 T-cells. To test whether MsrB1 influences DC-induced CD4 T-cell proliferation and activation in vitro, we isolated OT-II transgenic CD4 T-cells, which express OVA_323-339_-specific MHC II-restricted T-cell receptors, and cocultured them with OVA-pulsed WT and MsrB1-deficient BMDCs. Indeed, the absence of MsrB1 in the DCs reduced OT-II cell proliferation (Figure 2A) and their upregulation of CD25 and CD44, which are T-cell activation markers (Figure 2B). The impaired T-cell stimulation was not due to the reduced viability of the MsrB1-deficient BMDCs in the coculture (Figure S3A). Moreover, OT-II cell death was not affected by the absence of MsrB1 in BMDCs; this suggests that MsrB1 in antigen-presenting cells may support the priming and activation of T-cells rather than their survival (Figure S3B). Notably, the absence of MsrB1 had no effect on the ability of OVA-pulsed BMDCs to induce the proliferation and activation of OT-I cells, which are transgenic OVA_257-264_-specific MHC class I-restricted CD8 T-cells (Figure S4). These observations together suggest that MsrB1 in DCs promotes the ability of these cells to present antigen on MHC class II and to express the costimulatory molecules needed to activate CD4 T-cells.

### 3.3. MsrB1 Is Needed for STAT6 Activation in BMDCs

**Figure 3:**
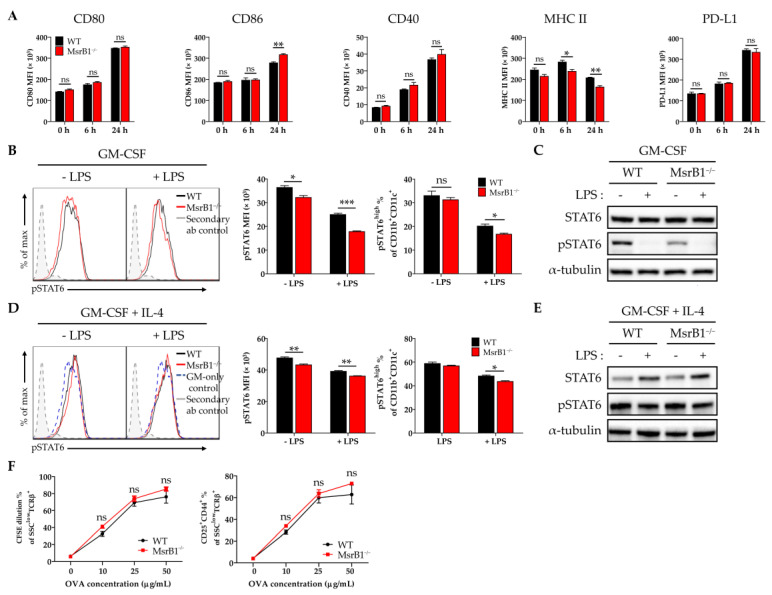
The ability of MsrB1 to control BMDC maturation and T-cell activation associates with MsrB1 regulation of STAT6 activation. (**A**) WT and MsrB1^−/−^ BMDCs were generated with IL-4 and granulocyte-macrophage colony-stimulating factor (GM-CSF) and treated with LPS for the indicated durations (*n* = 3 per group). Their expression of CD80, CD86, CD40, MHC II, and PD-L1 was then analyzed by flow cytometry. (**B**–**E**) GM-CSF-generated BMDCs (**B**,**C**) and IL-4/GM-CSF-generated BMDCs (**D**,**E**) were stimulated with LPS for 0 or 30 min (*n* = 3 per group) and their phosphorylated STAT6 levels were determined by flow cytometry (**B**,**D**) and Western blot (**C**,**E**). The frequencies of pSTAT-positive activated BMDC populations were determined by gating on the CD11c^+^CD11b^+^ cells in the live cells. (**F**) WT and MsrB1^−/−^ IL-4/GM-CSF-generated BMDCs were loaded with 0, 10, 25, or 50 μg/mL OVA and then cocultured for 3 days with CFSE-stained OT-II cells (*n* = 3 per group), after which the CFSE dilution and CD25^+^CD44^+^ frequencies of the T-cells were measured by flow cytometry by gating on the SSC^low^TCRβ^+^ cells in the live cells. Mean ± SEM are shown. * *p* < 0.05, ** *p* < 0.01, *** *p* < 0.001; ns, not significant, as determined by unpaired *t-*test. The data are representative of two (**B**,**D**,**F**), three (**C**), or four (**A**) independent experiments, all of which had similar results.

The experiments in Figure 1 that showed MsrB1 deficiency downregulates LPS-induced DC maturation were conducted with BMDCs that were generated with GM-CSF alone. Since the in vitro treatment of bone marrow progenitors with IL-4 as well as GM-CSF generates phenotypically different BMDCs [24], we also examined the effect of MsrB1 deficiency on the development and function of BMDCs that had been generated with both IL-4 and GM-CSF. Unlike in GM-CSF-generated BMDCs (Figure 1A,B), MsrB1 deficiency did not reduce the LPS-induced costimulatory molecule expression of these DCs; indeed, these cells showed slightly higher LPS-induced CD86 expression compared to the WT DCs (Figure 3A). However, like GM-CSF-generated BMDCs (Figure 1D), the MsrB1 deficiency in IL-4/GM-CSF-generated BMDCs was associated with significantly lower MHC class II expression, although only when the cells were stimulated with LPS (Figure 3A). This difference between the IL-4/GM-CSF- and GM-CSF-generated BMDCs may reflect the fact that while GM-CSF maintains basal levels of STAT6 activation, thereby promoting DC maturation, this effect is potently augmented by adding IL-4 [25,26]. In other words, the greater ability of the IL-4/GM-CSF-generated BMDCs to resist the DC maturation-reducing effects of MsrB1 deficiency may reflect the presence of IL-4, which induced sufficient STAT6 activation to complete the DC expression of the costimulatory molecule [27].

These observations led us to hypothesize that MsrB1 may shape DC maturation and function by regulating the activation of the STAT6 pathway. To test this, we measured the phosphorylated STAT6 (pSTAT6) levels in BMDCs that had been generated with GM-CSF alone or with GM-CSF plus IL-4. LPS treatment reduced the upregulated pSTAT6 levels in both BMDC types, regardless of whether they were derived from WT or MsrB1-deficient mice. Notably, the absence of MsrB1 significantly reduced the pSTAT6 levels and frequency of pSTAT6^+^ cells in the GM-CSF-generated BMDCs, particularly after LPS stimulation (Figure 3B,C). By contrast, in the IL-4-/GM-CSF-generated BMDCs, the MsrB1 deficiency reduced pSTAT6 expression and the frequency of pSTAT6 positivity much less; only mild downregulation of pSTAT6 or pSTAT6 positivity was observed in the LPS-untreated and -treated cells (Figure 3D,E). These findings are consistent with the fact that MsrB1 deficiency had no significant effect on the ability of OVA-loaded IL-4/GM-CSF-generated BMDCs to stimulate OT-II cells (Figure 3F); this contrasts directly with the sharply deleterious effect of MsrB1 deficiency on the ability of GM-CSF-generated BMDCs to stimulate OT-II cells, as shown in Figure 2. These data suggest that MsrB1 may support the antigen presentation and T-cell priming functions of GM-CSF-generated BMDC by maintaining sufficient pSTAT6 levels in these cells.

### 3.4. MsrB1 Potentiates IL-12 Production by BMDC In Vitro

**Figure 4:**
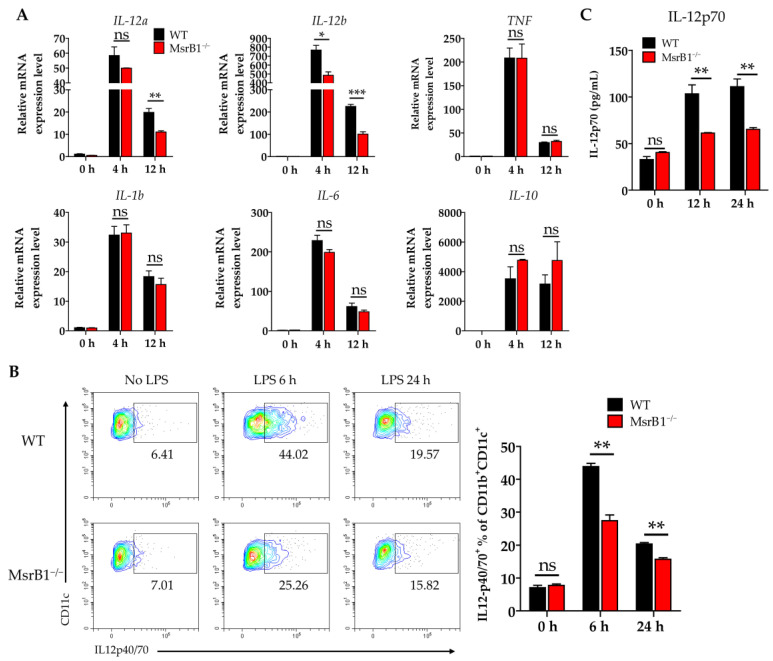
MsrB1 promotes the production of IL-12 by DCs in vitro. (**A**) WT and MsrB1^−/−^ BMDCs were stimulated with LPS for the indicated times (*n* = 3 per group) and their transcript levels of *IL-12a*, *IL-12b*, *TNF*, *IL-1b*, *IL-6*, and *IL-10* were analyzed by qRT-PCR. (**B**) The intracellular IL-12p40/70 levels in WT and MsrB1^−/−^ BMDCs at the indicated time points after LPS stimulation (*n* = 3 per group) were measured by flow cytometry with gating on the CD11c^+^CD11b^+^ cells in the live cells. (**C**) The extracellular levels of IL-12p70 in the BMDC cultures were measured by ELISA (*n* = 3 per group). Mean ± SEM are shown. * *p* < 0.05, ** *p* < 0.01, *** *p* < 0.001; ns, not significant, as determined by unpaired *t-*test. The data are representative of two experiments, which had similar results.

In addition to antigen presentation (signal 1) and expression of costimulatory ligands (signal 2), another major function of DCs as professional antigen-presenting cells is to provide T-cells with cytokine signaling (signal 3). We therefore examined whether MsrB1 shaped the cytokine production of LPS-treated BMDCs. When we analyzed several key DC cytokines, we found that MsrB1 deletion significantly downregulated the LPS-induced BMDC transcription of *IL-12a* and *IL-12b* at 12 h (Figure 4A). These genes encode IL-12p35 and IL-12p40, which form a proinflammatory heterodimeric Th1 cytokine IL-12p70. The MsrB1-deficient BMDCs also expressed lower LPS-induced IL-12 protein levels than the WT BMDCs at 6 and 12 h (Figure 4B). Consistent with these data, the significantly lower amount of IL-12p70 was detected in the culture of MsrB1-deficient BMDCs after LPS stimulation (Figure 4C). In addition, MsrB1 deficiency tended to increase (rather than decrease) the production of IL-10, which is an anti-inflammatory cytokine (Figure 4A). This suggests that MsrB1 may promote the proinflammatory properties of DCs but not their anti-inflammatory properties. Interestingly, the MsrB1-deficient BMDCs that were generated with IL-4 and GM-CSF also showed defective LPS-induced IL-12 production (Figure S5); however, this effect was less strong than in the GM-CSF-generated BMDCs (Figure 4). In conclusion, MsrB1 plays an important role in the ability of DCs to produce IL-12. Expression of IL-23, which is composed of IL-12p40 and IL-23p19, is not significantly affected by MsrB1 deletion in BMDCs (Figure S6A,B). Accordingly, IL-23p19 was detected in the culture of WT and MsrB1-deficient BMDCs at a comparable level (Figure S6C). Collectively, MsrB1 selectively regulates transcription and protein expression of IL-12, not IL-23.

### 3.5. MsrB1 Promotes the Production of IL-12 by Classical Splenic DCs on LPS Challenge In Vivo

**Figure 5:**
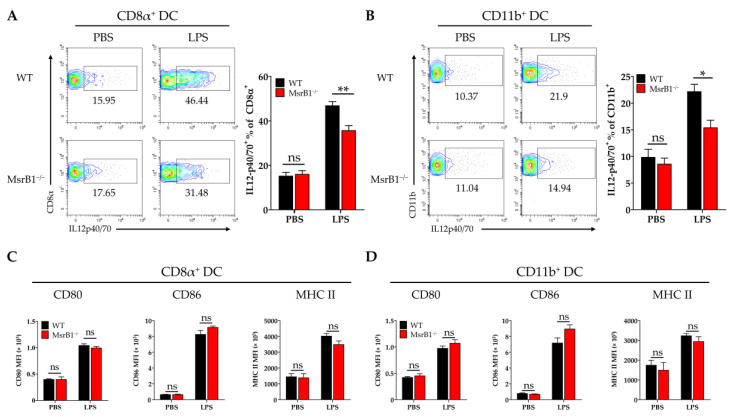
MsrB1 promotes the LPS-induced IL-12 production of classical splenic DCs in vivo. (**A**–**D**). Splenocytes isolated from WT and MsrB1^−/−^ mice 3 h after LPS injection were cultured in the presence of Brefeldin A for 4 h. The CD8α^+^ (**A**,**C**) and CD11b^+^ (**B**,**D**) DCs of the WT and MsrB1^−/−^ mice (*n* = 6 per group) were analyzed by flow cytometry for their intracellular IL-12 p40/70 levels (**A**,**C**) and their expression of CD80, CD86, and MHC II (**B**,**D**). The classical DCs were analyzed by gating on the CD8α^+^ or CD11b^+^ cells in the live CD11c^+^ MHC II^+^ cells. Mean ± SEM are shown. * *p* < 0.05, ** *p* < 0.01; ns, not significant, as determined by Mann-Whitney U test. The data are representative of two experiments, which had similar results.

To determine whether the effects of MsrB1 on DC functions in vitro are also observable in vivo, we challenged WT and MsrB1 knockout mice with LPS. The LPS challenge did not alter the phenotypic markers of the classical DC subsets in both WT and MsrB1-deficient mice (Figure S7). Next, in separate experiments, we obtained the splenocytes from LPS- and PBS-challenged WT and MsrB1-deficient mice and evaluated their expression of IL-12 and costimulatory molecules by the CD8α^+^ type I and CD11b^+^ type II classical DCs in the spleen. As expected, LPS induced both DC subsets to upregulate their expression of IL-12 but the CD8α^+^ DC produced more IL-12 than the CD11b^+^ DC. Nevertheless, both DC populations expressed less IL-12 when they came from the MsrB1 knockout mice rather than the WT mice (Figure 5A,B). In contrast, while LPS also caused both DC subsets to upregulate their costimulatory molecule and MHC class II expression, MsrB1 deficiency had no effect on these expression levels (Figure 5C,D). Thus, MsrB1 activity is required for the LPS-induced IL-12 production of splenic DC in vivo but is not needed for splenic DC maturation in vivo. In addition, MsrB1 deletion did not significantly affect IL-23 expression in both splenic DC subsets (Figure S6D).

### 3.6. MsrB1 in DCs Promotes DC-Induced Differentiation of Th1 Cells

**Figure 6:**
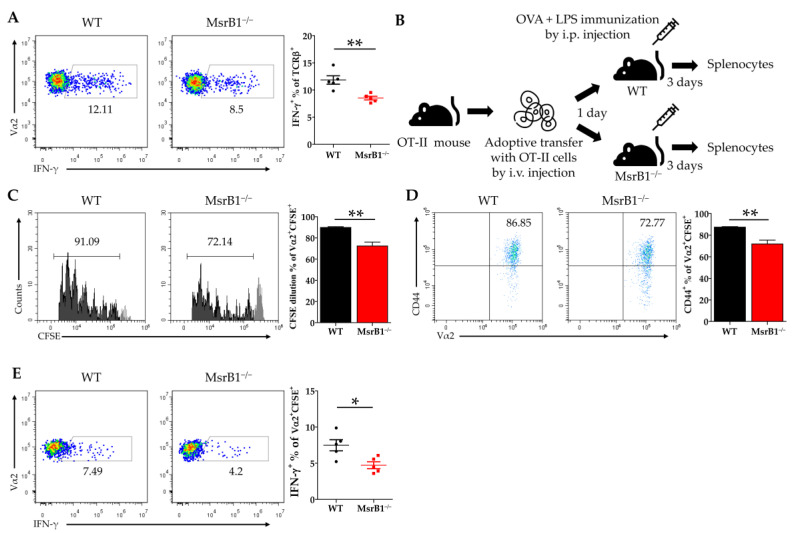
MsrB1 promotes the DC-induced differentiation of T-helper (Th)1 cells. (**A**) WT or MsrB1^−/−^ BMDCs were loaded with 50 μg/mL OVA and cocultured with OT-II cells for 3 days, after which the intracellular IFN-γ levels of the SSC^low^TCRβ^+^ OT-II cells were measured by flow cytometry (*n* = 3 per group). (**B**) Schematic depiction of the in vivo model that was used to assess the effect of MsrB1 deficiency on the ability of DCs to promote Th1 differentiation. Thus, 1 × 10^6^ CFSE-labeled CD4^+^ OT-II cells were adoptively transferred into WT and MsrB1^−/−^ mice, which were then injected intraperitoneally with OVA and LPS. Three days later, the OT-II cells in the spleens of the WT and MsrB1^−/−^ recipients (*n* = 5 per group) were assessed by flow cytometry for CFSE dilution (**C**), CD44^+^ expression (**D**), and intracellular IFN-γ expression (**E**). The transferred OT-II cell population was analyzed by gating on the Vα2^+^CFSE^+^ cells in the live CD3^+^CD4^+^ cells. Mean ± SEM are shown. * *p* < 0.05, ** *p* < 0.01; ns, not significant, as determined by Mann-Whitney U test. The data are representative of two experiments, which had similar results.

Since IL-12 is well-known to be a Th1 cell-promoting cytokine [28], our in vitro and in vivo data strongly suggest that MsrB1 in DCs may positively regulate the differentiation of Th1 cells from naïve CD4 T-cells by promoting DC secretion of IL-12. To test this, we first loaded WT and MsrB1-deficient BMDCs with OVA, cocultured them with OT-II cells, and measured the T-cell production of IFN-γ, the hallmark cytokine of Th1 cells. Indeed, MsrB1 deficiency was associated with less IFN-γ production (Figure 6A). Next, we investigated the physiological role of MsrB1 in Th1 differentiation in mice by intravenously transferring the naïve T-cells from OT-II mice into MsrB1-deficient and WT mice and then stimulating the mice with intraperitoneal injection of OVA and LPS (Figure 6B). Three days after the immunization, we assessed the activation, proliferation, and IFN-γ production of the transferred OT-II T-cells. When transferred into the MsrB1-deficient mice, the OT-II T-cells proliferated less well, expressed less CD44 (Figure 6C,D), and, most importantly, produced fewer IFN-γ-producing OT-II cells (Figure 6E). This confirms that MsrB1 supports Th1 differentiation in vivo. These data are consistent with the ability of MsrB1 to specifically regulate the DC production of IL-12 both in vivo and in vitro. Therefore, we conclude that MsrB1 may promote Th1 responses by upregulating the IL-12 production by DCs.

### 3.7. MsrB1 Deficiency Associates with Defective Differentiation of Tfh Cells In Vivo

**Figure 7:**
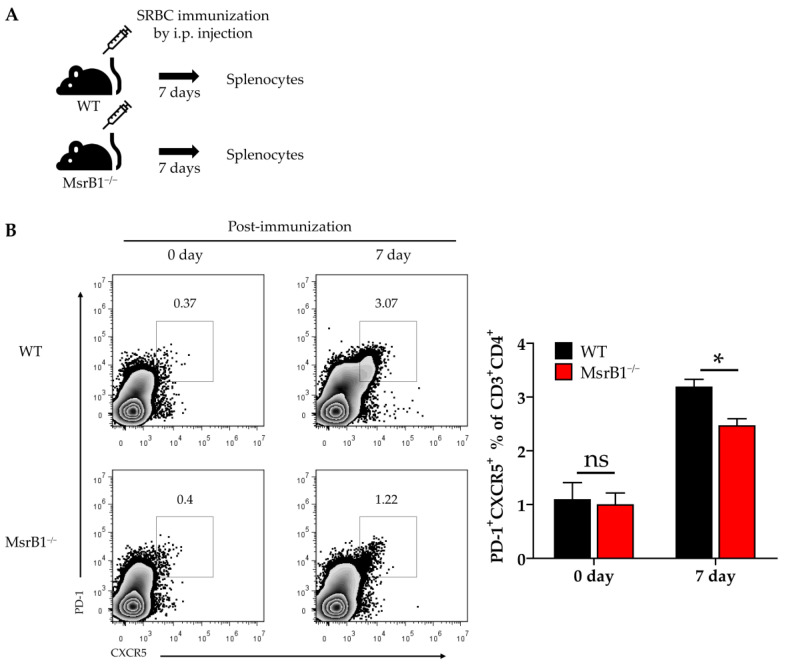
MsrB1 may promote follicular helper T-cell (Tfh) differentiation in vivo. (**A**) Schematic depiction of the sheep red blood cell (SRBC) immunization protocol. WT and MsrB1^−/−^ mice (*n* = 6 per group) were injected intraperitoneally with SRBC and the splenocytes were isolated 7 days later. (**B**) The Tfh (PD-1^high^CXCR5^high^ CD4^+^) cells in the spleen of the WT and MsrB1^−/−^ mice were measured by flow cytometry by gating on the CD3^+^CD4^+^ cells in the live cells. Mean ± SEM are shown. * *p* < 0.05; ns, not significant, as determined by the Mann-Whitney U test. The data are representative of two experiments, which had similar results.

We next sought to determine the physiological role of MsrB1 in eliciting other DC-regulated immune responses. When naïve CD4 T-cells engage with their antigen in the context of MHC Class II, they differentiate into several T-helper lineages depending on the cytokine environment. DC-derived IL-12 not only induces the differentiation of Th1 cells, it also plays an important role in the differentiation of follicular helper T (Tfh) cells [29,30]. Given the ability of MsrB1 to regulate DC production of IL-12, we speculated that MsrB1 may also promote the differentiation of Tfh cells in vivo. To test this, we immunized WT and MsrB1 knockout mice with SRBCs, which are known to induce a strong Tfh response [31]. A week after immunization, we evaluated the Tfh cell frequency in the spleen (Figure 7A) by identifying the CD4 T-cells that coexpressed PD-1 and C-X-C chemokine receptor type 5 (CXCR5) [32]. Compared to the WT mice, the MsrB1-deficient mice had significantly fewer splenic Tfh cells after SRBC immunization (Figure 7B). Thus, MsrB1 may participate in SRBC immunization-induced Tfh differentiation, perhaps by promoting IL-12 signaling. While it remains unclear whether the Tfh cell defect in MsrB1 knockout mice is a cell-intrinsic or -extrinsic effect, this result clearly shows that MsrB1 plays a physiologically relevant role in immunity.

## 4. Discussion

In this study, we proposed a novel concept: the selenoprotein MsrB1, which is known to modulate protein functions by reducing oxidized Met residues [33], may regulate adaptive immune responses by shaping the ability of DCs to present antigen and produce costimulatory molecules and cytokines. Our data also suggested that this activity of MsrB1 may be mediated by its effects on the STAT6 pathway in DCs and the IL-12 production of DCs. In addition, we found that MsrB1 may promote the immunization-induced differentiation of Th1 and Tfh cells.

The hugely diverse roles of ROS in normal and pathological immune responses have been studied for years. These roles include the use of ROS by innate immune cells to kill microbes [34], the activation of innate and adaptive immune cells by intracellular ROS that are induced by external signals [11,12,13], and the suppression of anticancer immune cells by ROS-producing myeloid-derived suppressor cells [35,36]. Thus, well-balanced levels of ROS/oxidation are critical for maintaining the line between immune health and pathology: while excessive ROS/oxidation is detrimental to immune cells, sufficient ROS/oxidation is needed to evoke effective immune responses. The fact that MsrB1 may be needed for the DC-induced activation of T-cells such as Th1 and Tfh cells and it contains selenium, an essential micronutrient whose deficiency associates with immune impairment [37], suggests that one feasible and practical way to maintain healthy ROS-mediated signaling in immune cells is diet intervention. Since selenium appears to protect against certain cancers [38] and pathogens [39], it may be worthwhile to study the role that MsrB1 plays in the immunological processes that are involved in cancer and pathogen challenge.

Intracellular signaling is shaped by a diverse range of post-translational protein modifications and the interplays between different modifications. Our finding that MsrB1 may shape the ability of DCs to activate T-cells by upregulating the major transcription factor STAT6 suggested that Met oxidation/reduction may control STAT6 activation. It remains unclear whether STAT6 is a direct target of MsrB1 or only indirectly associates with the MsrB1-mediated pathway. There is some limited evidence that STAT6 may be targeted directly by the reductase activity of MsrB1. Thus, when bone marrow-derived mast cells are stimulated with IL-4, STAT6 in the nucleus undergoes proteolytic cleavage and the cleaved form acts as a dominant-negative regulator of STAT6-mediated transcription [40,41]. However, mutation of the Met 686 residue (which lies near the tyrosine 641 residue that is phosphorylated during STAT6 activation) inhibits the proteolytic cleavage of STAT6 [40]. Thus, it is possible that oxidation of Met 686 in STAT6 and its reduction by MsrB1 could, respectively, reduce and promote STAT6 phosphorylation, thereby dictating DC functions. Therefore, we hypothesize that MsrB1 may directly regulate the phosphorylation of STAT6 and its transcriptional activity by inducing a conformational change in STAT6 (or by regulating STAT6 cleavage). However, it remains possible that MsrB1 contributes to STAT6 activation (or cleavage) only indirectly by reducing the oxidized Met residue(s) of unknown factors. Further experimentation is needed to determine whether the effect of MsrB1 deficiency on DC antigen presentation and T-cell activation is directly mediated by poor reduction of Met residues in STAT6 and the consequent lower STAT6 phosphorylation/activation.

It should be noted that we identified the upregulatory effect of MsrB1 on STAT6 phosphorylation by in vitro assays that compared BMDCs that had been generated from progenitors by GM-CSF alone and GM-CSF with IL-4. These data suggested that MsrB1 not only strongly controls the basal STAT6 activation induced by GM-CSF, it also controls, albeit more weakly, IL-4-induced STAT6 activation (Figure 3B–E). Nevertheless, the addition of IL-4, a strong STAT6 activator [42], to the GM-CSF-containing medium was sufficient to completely rescue the costimulatory ligand expression of the MsrB1-deficient BMDCs; it also largely improved their MHC class II expression (Figure 3A versus Figure 1A,B,D). Predictably, this effect of IL-4 also completely rescued the ability of the MsrB1-deficient BMDCs to promote T-cell activation (Figure 3F vs Figure 2).

Our study also showed that MsrB1 deficiency not only reduced pSTAT6 levels, it also decreased their IL-12 production and surface expression of costimulatory molecules. Interestingly, these effects, which were both quite strong in the GM-CSF-generated BMDCs, were comparatively weaker in the IL-4/GM-CSF-generated BMDCs (Figure 3A, Figure S5). This may be explained by a recent study showing that IL-4 promotes IL-12 expression in BMDCs in a STAT6-dependent manner [43]. Thus, while IL-4-mediated STAT6 phosphorylation in DCs may be weakly controlled by MsrB1, there are also other non-MsrB1-related mechanisms that regulate the effect of IL-4 on STAT6 in DCs. Our data showed that the latter mechanisms compensated for the lack of MsrB1 in the IL-4/GM-CSF-induced BMDCs: they induced enough STAT6 phosphorylation to produce adequate IL-12 levels and costimulatory molecules.

Our finding that MsrB1 controlled the IL-12 production of DCs led us to speculate that by shaping DC antigen presentation functions, MsrB1 also controls T-cell differentiation. Specifically, we considered it possible that MsrB1 promotes Th1 and Tfh differentiation, which are both strongly driven by IL-12. Indeed, we observed that adoptively transferred OVA-specific T-cells were significantly more likely to adopt a Th1 (IFN-γ-expressing) phenotype in WT mice than in MsrB1-deficient mice. Moreover, SRBC injections strongly upregulated the splenic frequency of Tfh cells in WT but not MsrB1-deficient mice. To our knowledge, this is the first time that it has been shown that MsrB1-mediated Met Redox controls T-cell differentiation.

It should be noted, however, that while MsrB1 deficiency was associated with less IL-12 expression and Th1 polarization both in vitro and in vivo, the ability of MsrB1 deficiency to reduce DC maturation was only observed when we used GM-CSF-generated BMDCs in vitro (Figure 1); this effect of MsrB1 was not observed in vivo (Figure 5C,D). This apparent discrepancy may reflect the presence of compensatory selenoproteins in the splenic DCs or another in vivo signaling source that can replace GM-CSF-induced signal transduction. It should also be noted that mammalian cells express three additional methionine sulfoxide reductases other than MsrB1, namely, MsrA, MsrB2, and MsrB3. These proteins are expressed in different cell types [6]. Gene expression profiling of all methionine sulfoxide reductases will give more insight into the mechanisms by which these proteins control adaptive immune responses.

In this study, we primarily focused on the role of MsrB1 in DC antigen presentation and T-cell priming. It is entirely possible that MsrB1 also participates in other immune cell processes, including T-cell activation. This is supported by the fact that (i) T-cells express ROS when their T-cell receptors engage with antigen-bearing MHC molecules [10] and (ii) an analysis of mice that lacked the ability to produce Sec residues in T-cells indicated that endogenous selenoproteins expressed in T-cells participate in T-cell activation [44]. MsrB1 may also control macrophage immune activities: MsrB1 ablation in bone marrow-derived macrophages promote their M-CSF-induced production of anti-inflammatory cytokines [17]. The ability of MsrB1 to stimulate the production of proinflammatory cytokines in DCs while driving anti-inflammatory cytokine production in macrophages may reflect the fact that GM-CSF and M-CSF, respectively, induce pro- and anti-inflammatory functions in myeloid cells [45]. These observations suggest that MsrB1 may have different effects depending on the target cell type and local conditions. It will be of interest to study how the functions of MsrB1 in T-cells, antigen-presenting cells, and other cell types in specific immune microenvironments coordinate to elicit effective immune responses. Moreover, it may be illuminating to study the differential roles that MsrB1 plays in different immune cell subsets at various stages of particular immune responses.

## 5. Conclusions

We showed that MsrB1 in DCs, which are professional antigen-presenting cells, appears to promote DC-mediated T-cell priming and Th1 differentiation. Our study sheds new light on the ability of Met reduction to regulate signal transduction and thereby shape immunity. These findings suggest that Met Redox mechanisms could be a therapeutic target whose manipulation may induce the recovery of or promote healthy immune responses.

## Figures and Tables

**Table 1:** Quantitative real-time PCR (qPCR) primers used in this study.

Gene	Accession Number	Forward Primer (5′ to 3′)	Reverse Primer (5′ to 3′)
*TNF*	NM_013693	ACAGAAAGCATGATCCGCG	GCCCCCCATCTTTTGGG
*IL-1b*	NM_008361.4	GTGGCTGTGGAGAAGCTGTG	GAAGGTCCACGGGAAAGACAC
*IL-6*	NM_031168.2	CCAGAAACCGCTATGAAGTTCC	TTGTCACCAGCATCAGTCCC
*IL-10*	NM_010548	ACACCTTGGTCTTGGAGCTT	AGAGAAGCATGGCCCAGAAA
*IL-12a*	NM_008351.3	CAGAAACCTCCTGTGGGAGA	GGAGCTCAGATAGCCCATCA
*IL-12b*	NM_001303244.1	ATCCAGCGCAAGAAAGAAAA	GGAACGCACCTTTCTGGTTA
*Il-23a*	NM_031252.2	CCTCTCCGTTCCAAGATCCT	ACTAAGGGCTCAGTCAGAGTTGCT
*Ppia*	NM_008907.1	ATGGTCAACCCCACCGTGT	TTCTTGCTGTCTTTGGAACTTTGTC

**Table 2:** FACS antibodies used in this study.

Antigen	Conjugate	Clone	Company	Catalog
CD3	PE/Cy7	145-2C11	BioLegend	100320
CD4	Brilliant violet605	GK1.5	BioLegend	100451
CD4	FITC	GK1.5	BioLegend	100406
CD8a	PE/Cy7	53-6.7	BioLegend	100714
CD11b	APC	M1/70	BioLegend	101212
CD11c	PE/Cy7	N418	BioLegend	117318
CD11c	Alexa700	N418	BioLegend	117319
CD25	PE/Cy7	PC61	BioLegend	102016
CD40	APC	3/23	BioLegend	124612
CD44	APC	IM7	BioLegend	103012
CD80	Pacific blue	16-10A1	BioLegend	104724
CD80	Brilliant violet421	16-10A1	BioLegend	104726
CD86	PE	GL-1	BioLegend	105008
I-A/I-E	FITC	M5/114.15.2	BioLegend	107605
TCRβ	PE	H57-597	BioLegend	109208
TCRVα2	PE	B20.1	BioLegend	127808
CXCR5	PE	L138D7	BioLegend	145504
PD-1	APC	J43	eBioscience	17998582
PD-L1	PE	10F.9G2	BioLegend	124308
IL-12p40/70	APC	C15.6	BD Pharmingen	554480
IFN-γ	PE/Cy7	XMG1.2	BioLegend	505826
IL-23 p19	eFluor 660	fc23cpg	eBioscience	50-7023-82
Rabbit IgG	Alexa647		Invitrogen	A32733

## Supplementary Materials

The following are available online at https://www.mdpi.com/2076-3921/9/10/1021/s1, Figure S1: Bone marrow-derived DCs express MsrB1 and MsrB1 deficiency do not affect classical DC differentiation in the spleen; Figure S2: MsrB1 deficiency does not affect the GM-CSF-induced differentiation of BMDCs from bone marrow progenitors; Figure S3: MsrB1 deficiency does not affect OVA-loaded BMDC or T-cell survival; Figure S4: MsrB1^−/−^ BMDCs present antigen normally to CD8+ T-cells; Figure S5: MsrB1-deficient BMDCs generated with IL-4 and GM-CSF showed decreased LPS-induced IL-12 production in vitro; Figure S6: MsrB1 does not regulate LPS-induced IL-23 production in DCs; Figure S7: MsrB1 deficiency does not change the effect of LPS challenge on classical DC subsets in the spleen.
