# Supplementary material for: The Selenoprotein MsrB1 Instructs Dendritic Cells to Induce T-Helper 1 Immune Responses
Source: Antioxidants (Basel). 2020 Oct 20;9(10):1021. doi: 10.3390/antiox9101021 (PMC7589095; doi:10.3390/antiox9101021)
Supplement: Supplementary file 1 [file antioxidants-09-01021-s001.pdf]

Supplementary Figures and legends

A

B

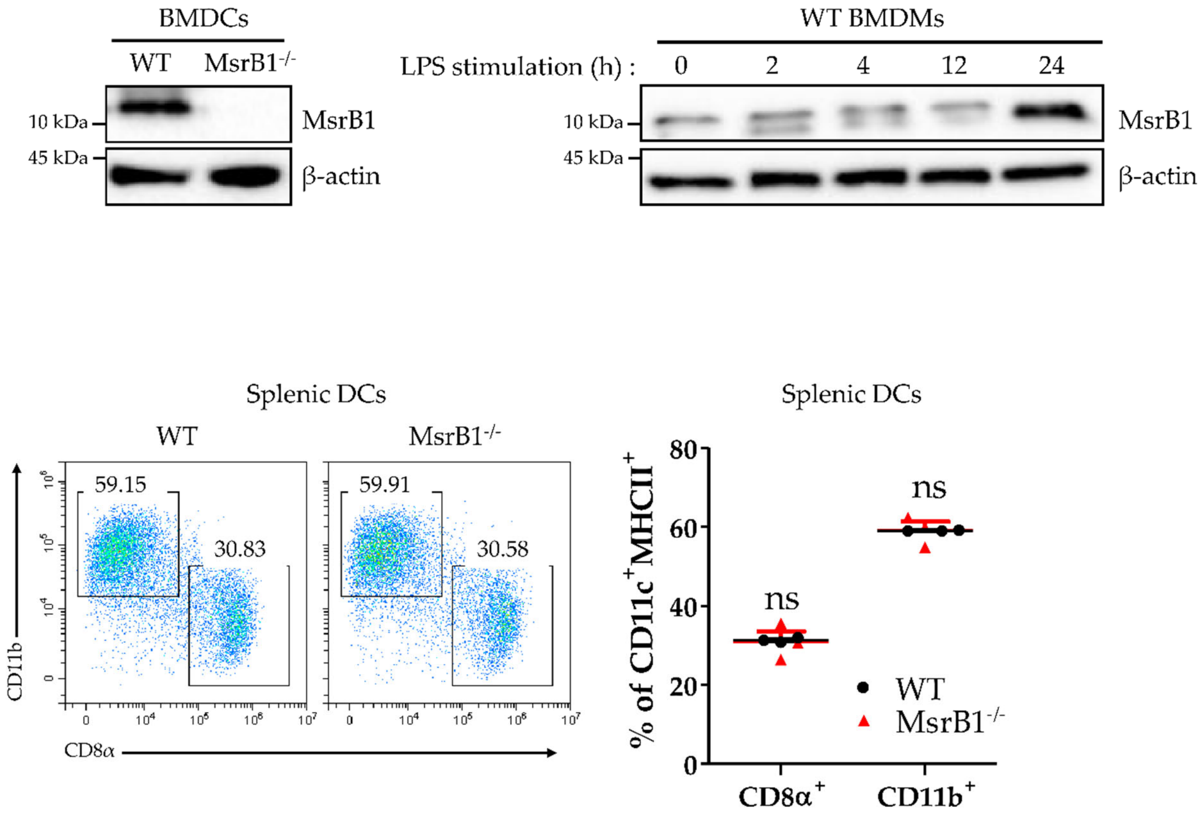

Supplementary Figure 1. Bone marrow-derived DCs express MsrB1 and MsrB1 deficiency does not affect classical DC differentiation in the spleen. (A) Bone marrow-derived DCs (BMDCs, left) and macrophages (BMDMs, right) were generated from WT and MsrB1$^{-/-}$ mice and then subjected on day 8 of culture to western blotting of MsrB1 expression. β-actin was used as housekeeping control. (B) The classical DCs in the spleen of WT and MsrB1$^{-/-}$ mice (*n* = 3 per group) were analyzed by flow cytometry by gating on the CD11c^{hgih}MHC II^{high} cells in the live cells. Mean ± SEM are shown. The data shown are representative of two independent experiments that had similar results. ns, not significant, as determined by unpaired *t*-test.

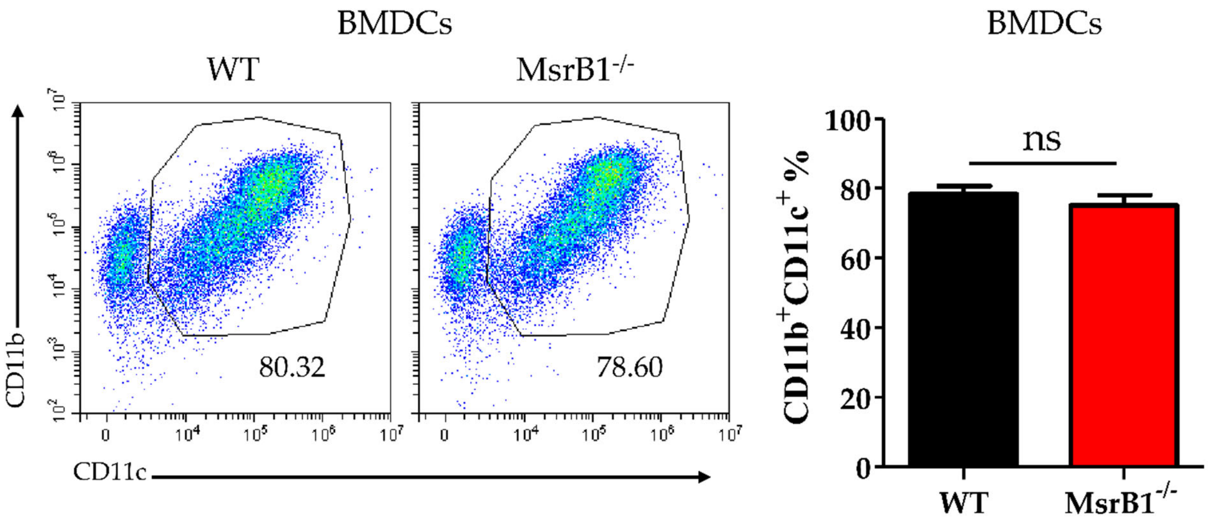

Supplementary Figure 2. MsrB1 deficiency does not affect the GMCSF-induced differentiation of BMDCs from bone-marrow progenitors. BMDCs were generated from WT and MsrB1$^{-/-}$ mice (*n* = 3 per group) with GMCSF and then analyzed on day 8 of culture by flow cytometry with gating on the CD11c^{hgih}CD11b^{high} cells in the live cells. Mean ± SEM are shown. The data shown are representative of four independent experiments that had similar results. ns, not significant, as determined by unpaired *t*-test.

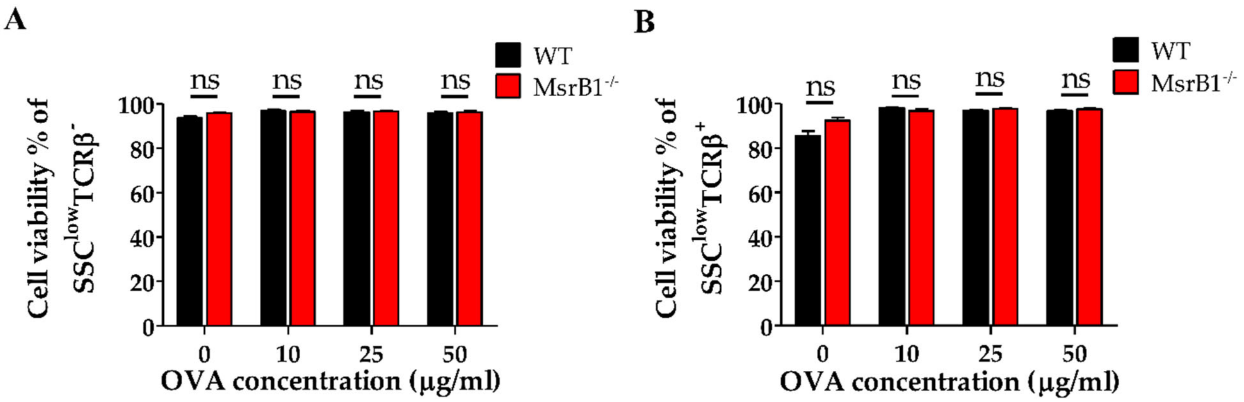

Supplementary Figure 3. MsrB1 deficiency does not affect OVA-loaded BMDC or T-cell survival. WT and MsrB1$^{-/-}$ BMDCs were loaded with 0, 10, 25, or 50 μg/ml OVA and co-cultured with naïve OT-II cells (*n* = 3 per group). The co-cultures were subjected to flow cytometry to measure the frequency of propidium iodide (PI)-negative BMDCs (A) and OT-II cells (B). The BMDC populations were analyzed by gating on the SSC^{low}TCRβ^{-} in the live cells. The OT-II cell populations were analyzed by gating on the SSC^{low}TCRβ^{+} cells in the live cells. Mean ± SEM are shown. The data shown are representative of two independent experiments that had similar results. ns, not significant, as determined by unpaired *t*-test.

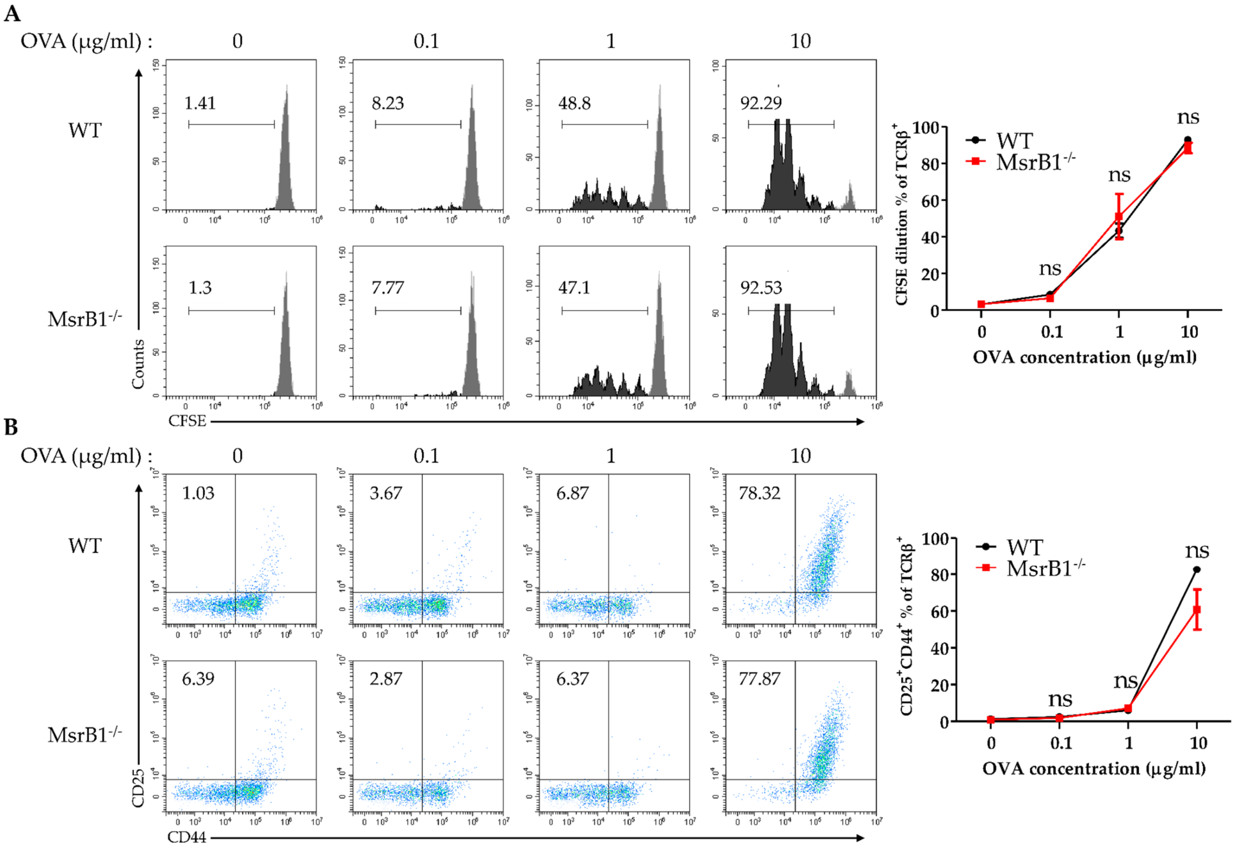

Supplementary Figure 4. MsrB1-/- BMDCs present antigen normally to $CD8^+$ T cells. Naïve OT-I cells, which are transgenic $OVA_{257-264}$-specific MHC class I-restricted CD8 T cells, were stained with CFSE and co-cultured for 3 days with WT and MsrB1-/- BMDCs that had been loaded with 0, 0.1, 1, or 10 µg/ml OVA (*n* = 3 per group). CFSE dilution (A) and OT-I cell activation, as measured by $CD25^{high}CD44^{high}$ cell frequencies (B) were measured by flow cytometry by gating on the $SSC^{low}TCR\beta^+$ cells in the live cells. Mean ± SEM are shown. The data shown are representative of two independent experiments that had similar results. ns, not significant, as determined by unpaired *t*-test.

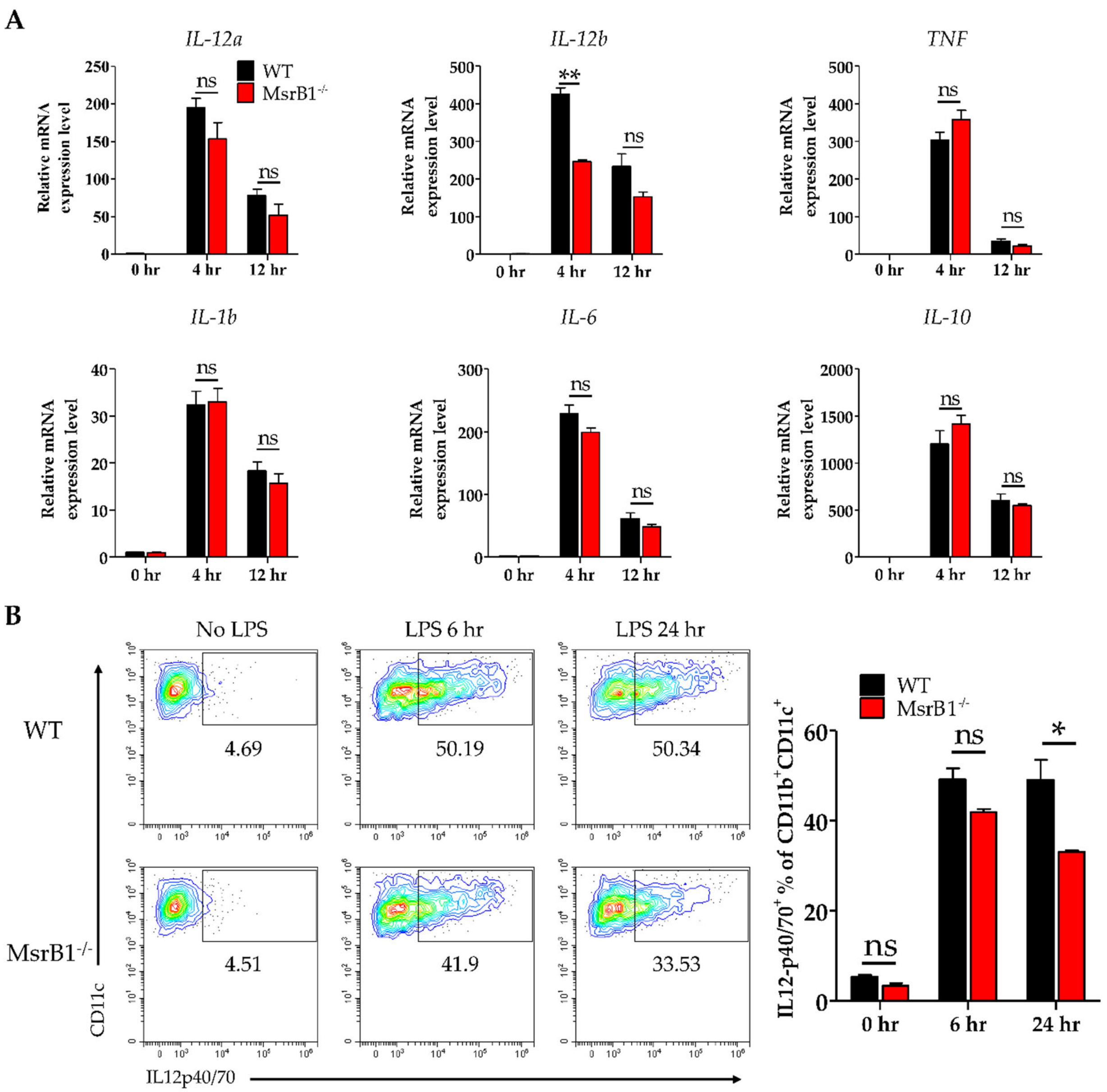

Supplementary Figure 5. MsrB1-deficient BMDCs generated with IL-4 and GM-CSF showed decreased LPS-induced IL-12 production *in vitro*. WT and MsrB1-/- BMDCs were generated with IL-4 and GM-CSF and treated with LPS for the indicated time (n = 3 per group). (A) qRT-PCR was used to measure the BMDC transcript levels of *IL-12a*, *IL-12b*, *TNF*, *IL-1b*, *IL-6*, and *IL-10*. (B) Flow cytometry with gating on the CD11c^{high}CD11b^{high} cells in the live cells was used to measure the intracellular BMDM levels of IL-12p40/70 levels. Mean ± SEM are shown. The data shown are representative of two independent experiments that had similar results. *, $P < 0.05$; ns, not significant, as determined by unpaired t-test.

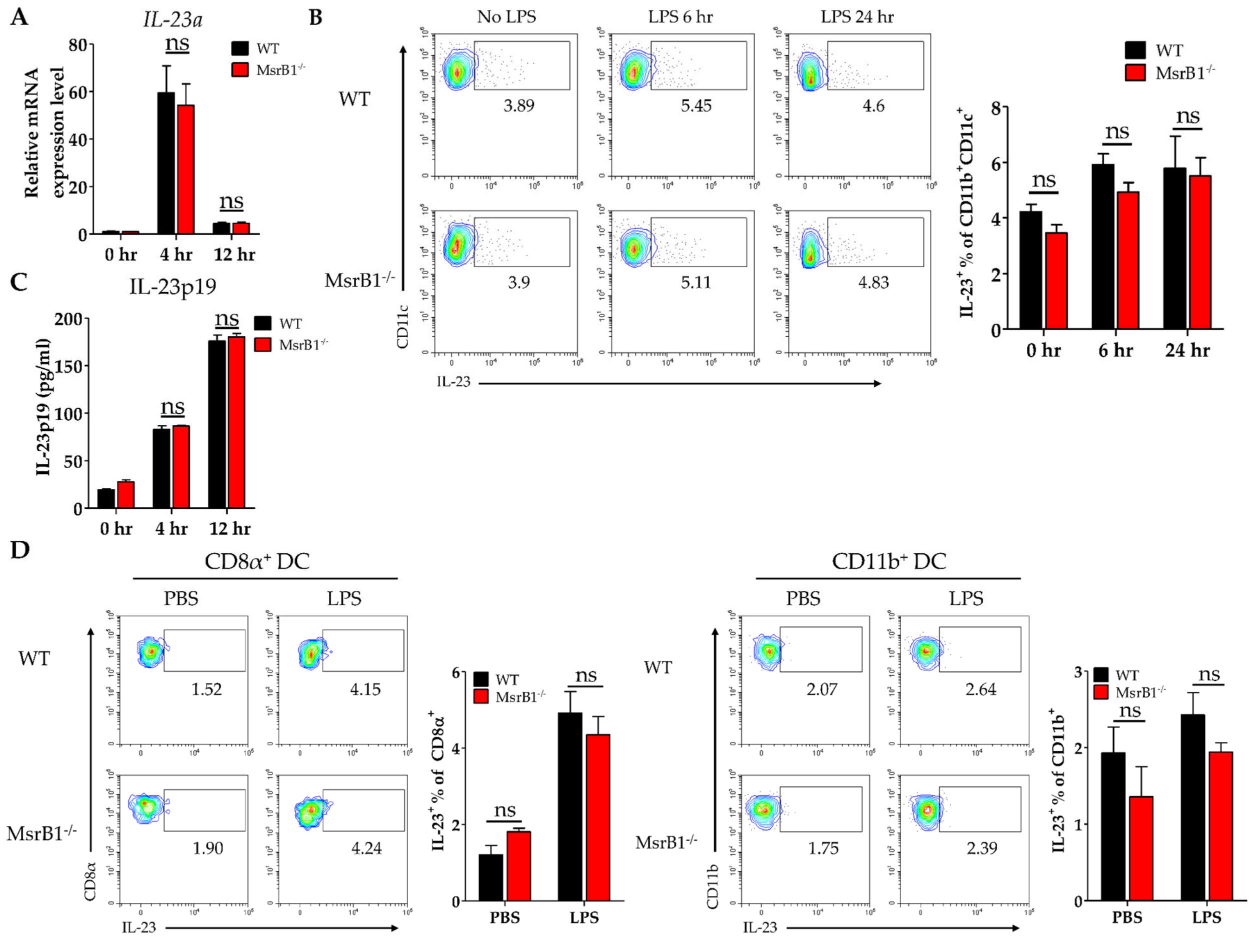

Supplementary Figure 6. MsrB1-deficient BMDCs showed normal LPS-induced IL-23 production. (A, B) WT and MsrB1$^{-/-}$ BMDCs were generated with GM-CSF and treated with LPS for the indicated time (*n* = 3 per group). (A) qRT-PCR was used to measure the BMDC transcript levels of *IL-23a*. (B) Flow cytometry with gating on the CD11c^{high}CD11b^{high} cells in the live cells was used to measure the intracellular BMDM levels of IL-23 levels. (C) The levels of IL-23p19 in the culture media were measured by ELISA (D) Splenocytes isolated from WT and MsrB1$^{-/-}$ mice 3 hr after LPS injection were cultured in the presence of Brefeldin A for 4 hr. The CD8α^{+} (left) and CD11b^{+} (right) DCs of the WT and MsrB1$^{-/-}$ mice (*n* = 3 per group) were analyzed by flow cytometry for their intracellular IL-23 levels. The classical DCs were analyzed by gating on the CD8α^{+} or CD11b^{+} cells in the live CD11c^{+} MHC II^{+} cells. Mean ± SEM are shown. The data shown are representative of two independent experiments that had similar results. ns, not significant, as determined by unpaired *t*-test.

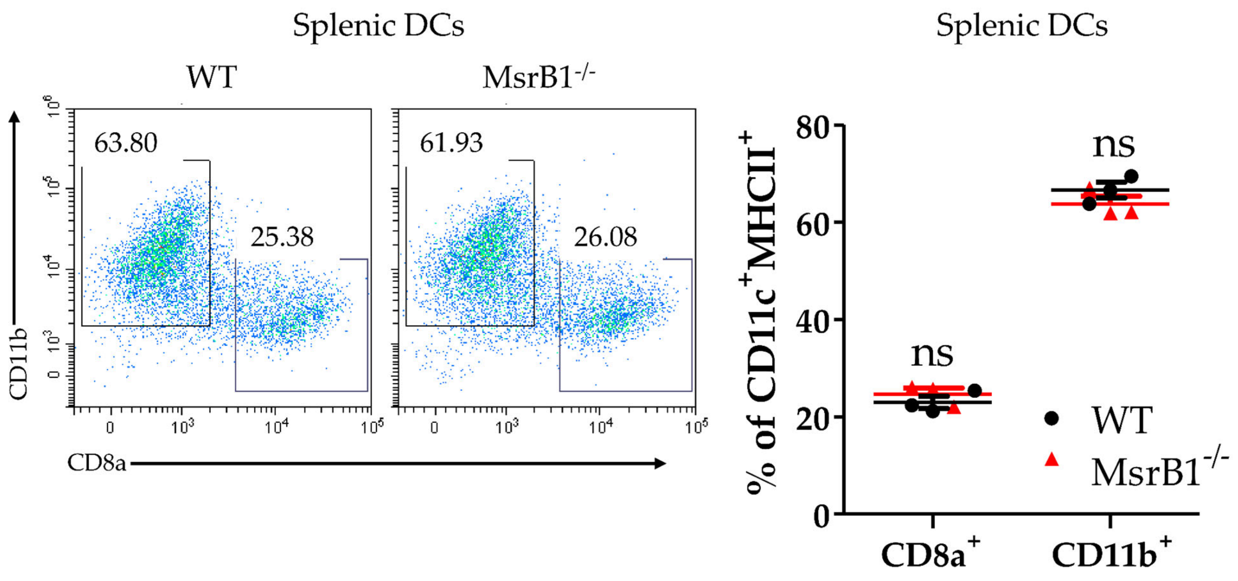

Supplementary Figure 7. MsrB1 deficiency does not change the effect of LPS challenge on classical DC subsets in the spleen. WT and MsrB1$^{-/-}$ mice were injected intraperitoneally with LPS. Three hours later, the splenocytes were harvested and the CD8α^{+} and CD11b^{+} cells were counted by flow cytometry using gating on the CD11c^{+}MHC II^{+} cells in the live cells. Mean ± SEM are shown. ns, not significant, as determined by unpaired *t*-test. The data shown are representative of two experiments that had similar results.
